# BRAF V600E Mutant Lung Adenocarcinoma Presenting With a Skull Base Metastasis and Pituitary Adenoma Collision Tumour

**DOI:** 10.7759/cureus.18180

**Published:** 2021-09-21

**Authors:** Sophie Heritage, Dominic O'Donovan, Tilak Das, Richard Mannion, Venkata R Bulusu

**Affiliations:** 1 Oncology, Cambridge University Hospitals, Cambridge, GBR; 2 Histopathology, Cambridge University Hospitals, Cambridge, GBR; 3 Radiology, Cambridge University Hospitals, Cambridge, GBR; 4 Neurosurgery, Addenbrooke's Hospital, Cambridge University Hospitals NHS Foundation Trust, Cambridge, GBR; 5 Primrose Oncology Unit, Bedford Hospital, Bedford, GBR

**Keywords:** adenocarcinoma lung, braf v600e mutation, collision tumour, pituitary adenoma, endoscopic transnasal approach

## Abstract

We report an unusual initial presentation for metastatic lung adenocarcinoma (LAC) with progressive loss of vision, a rare molecular phenotype and rapid visual response to surgical resection. A 60-year-old female presented with rapid and progressive visual loss over four weeks. Contrast-enhanced CT and MRI scans showed an enhancing lobulated mass in the base of skull infiltrating into the sella turcica. The patient underwent transnasal endoscopic debulking of the mass with rapid improvement in her vision. Histology showed a collision tumour with a pituitary adenoma and a microacinar metastatic adenocarcinoma. Staging CT of the chest, abdomen and pelvis showed a T4 N2 M1 right LAC. Molecular profiling of the metastasis confirmed an activating mutation involving codon 600 of BRAF gene (BRAF V600E). The patient was treated with combination chemotherapy but rapidly deteriorated and unfortunately died due to progressive disease. Efforts to access BRAF/MEK inhibitors for off-label use were unsuccessful. We believe our patient would have benefited from a BRAF/MEK inhibitor. This case illustrates the very unusual presentation of metastatic LAC with visual loss secondary to a collision tumour containing a pituitary adenoma and metastatic adenocarcinoma.

## Introduction

Lung cancer is a heterogeneous disease with different molecular subtypes. A subset of lung adenocarcinomas (LAC) have oncogenic driver mutations, including EGFR, ALK, ROS-1 and BRAF [1]. BRAF oncogenic driver mutations in LAC are rare, with a reported incidence of 1%-5% in Caucasian patients [1,2]. Collision tumours are two independent, histologically distinct tumours that occupy the same anatomical space [3]. Collision tumours of the sella turcica involving metastasis from lung cancer with co-existent pituitary adenomas are rare and we could only find one case report of this occurrence [4]. We report an unusual presentation of a rare subtype of LAC with BRAF mutation presenting with base of skull metastasis coexisting as a collision tumour with a pituitary adenoma.

## Case presentation

**Figure 1:**
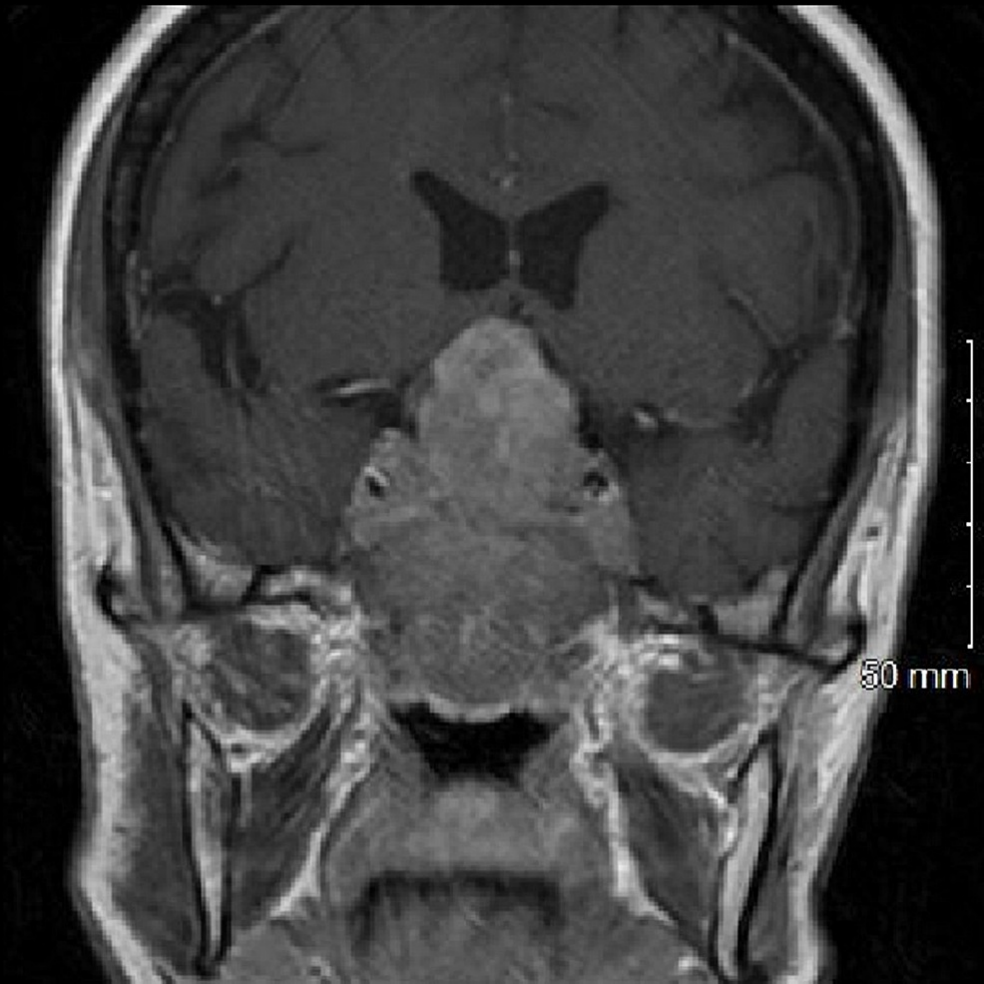
Post-contrast T1-weighted coronal reformat: a large enhancing mass has replaced the clivus and pituitary fossa, involving the posterior ethmoid region, nasopharynx and suprasellar cistern as well as the cavernous sinus bilaterally.

A 60-year-old female, a current smoker, presented with a four-week history of frontal headaches and deteriorating vision. Physical examination revealed anosmia, bilateral visual field deficits and reduced visual acuity. Contrast-enhanced CT and MRI head showed a 68 x 60 x 47 mm lobulated enhancing mass in the posterior ethmoid region, filling the nasopharynx. Intracranially, the mass replaced the clivus and pituitary fossa, extended into the cavernous sinus and suprasellar cistern and indented the inferior frontal lobes (Figure 1). Contrast-enhanced CT imaging of the chest, abdomen and pelvis showed a right-sided lung tumour with nodal involvement and no other visceral or bone metastases. The patient was transferred to the regional specialist centre for further management.

The patient underwent transnasal endoscopic resection of the tumour with optic chiasm decompression. A binostril endoscopic technique was used. The tumour was debulked using curettes, suction and microdebrider. This was a complex procedure and requiring careful identification of the optic nerves, chiasm, carotid vessels and the dura. Posterior septectomy and sphenoidotomy allowed for further tumour debulking. The tumour was then followed into the sella and was debulked until the diaphragma sella descended. The tumour was removed from the clival dura, optic chiasm and both optic nerves were cleared of the tumour.

**Figure 2:**
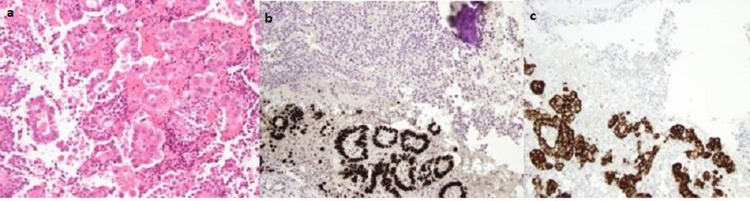
Histology and immunohistochemistry of base of skull metastasis: a: H&E showing microacinar adenocarcinoma; b: TTF1 +ve; c: BRAF-V600E +ve.

Histopathological assessment showed a very unusual lesion comprising of biphasic histology, metastatic microacinar adenocarcinoma colliding with a non-secretory gonadotroph pituitary neuroendocrine tumour (PitNET). The micro acinar adenocarcinoma cells were TTF1, AE1/AE3, CK7 and p53 positive (Figure 2). AFP, b-HCG, ER and melan-a were all negative. The PitNET was diffusely chromogranin positive but negative for all other pituitary endocrine markers.

Molecular phenotyping of the adenocarcinoma identified a missense mutation in the BRAF gene (Val600Glu) with nucleotide change of 1799 T>A. In addition, a missense mutation, (Ser37Ph-110C>T) in CTNNB1 gene was also identified. The adenocarcinoma was EGFR wildtype and showed no translocation/fusion of ALK and ROS-1. ROS-1 interphase FISH (fluorescence in-situ hybridisation) showed no rearrangement, but a gain of copy of ROS1. PD-L1 expression was 1%.

The patient rapidly regained her vision following the debulking and was able to function unaided. Baseline serum thyroid-stimulating hormone level was low at presentation but quickly normalised following the operation. Restaging CT scan of the chest, abdomen and pelvis showed progressive right-sided lung cancer with mediastinal nodes and pulmonary metastases, staged T4 N3 M1b. The patient was commenced on palliative chemotherapy but unfortunately the patient’s disease progressed and she passed away nearly 6 months after her debulking surgery. Her visual function was preserved. Efforts to obtain off-label BRAF/MEK inhibitors were not successful.

## Discussion

In most lung cancer patients, initial presentation is due to signs and symptoms from the primary lung cancer (dyspnoea, chest pain, persistent cough), or, less frequently, from metastasis to other parts of the body such as lymph nodes or bone. There have been some case reports of previously well patients presenting with visual disturbance as the initial presentation of metastatic LAC, due to pituitary metastasis [5]. However, in our case report, the patient’s metastasis was to the base of skull and extended into the sella turcica.

Collision tumours are two independent, distinct tumours that occupy the same anatomical space and are a rare subset of tumours, especially when located in the sella turcica [3,4]. Pituitary collision tumours can be surgically challenging due to the limited space for operation and biopsy, in addition to the predilection of the tumours to complicate surgical resection by adhering to surrounding structures [3]. The Endoscopic trans sphenoidal resection approach has multiple advantages, including reduced risk of developing postoperative diabetes insipidus, a common post-operative complication [3]. In addition, it is associated with greater gross tumour removal and decreased incidence of septal perforation [3]. Since this patient’s initial presenting complaint was visual disturbance, it is relevant that for pituitary macroadenomas, endoscopic endonasal surgery has been associated with good success rates for vision recovery after surgery [6]. In this case, the patient’s vision rapidly improved following surgery, highlighting the importance of considering specialist debulking surgery to alleviate the pressure symptoms related to a sellar mass.

The tumour was also found to be BRAF V600E mutant. The BRAF gene encodes a cytosolic serine/threonine-protein kinase that is involved in controlling cell growth and proliferation [1,2]. BRAF mutations are seen in 1%-5% of non-small cell lung cancer (NSCLC) patients, with V600E accounting for 50% of these [1]. BRAF mutations are more frequent in females and current or former smoking patients. [1]. These mutations result in activation of the MAPK pathway and unregulated cell growth, proliferation and survival [1].

BRAF-mutated lung cancers tend to be more aggressive and resistant to chemotherapy, but targeted tyrosine kinase inhibitors such as BRAF inhibitors (in combination with MEK inhibitors) can be effective [1,7]. The safety and efficacy of the BRAF inhibitor dabrafenib in combination with MEK inhibitor trametinib have been evaluated in a phase 2 study in patients with BRAF V600E-mutant NSCLC [7]. Other reports have also shown benefit of BRAF/MEK inhibition, ranging from partial to complete response, in BRAFV600E-mutant LAC with intracranial metastatic lesions [8]. Our patient may have benefited from the dual BRAF+MEK inhibitor combination. It has been shown that the BRAF inhibitor vemurafenib, approved for use in metastatic melanoma, can cross the blood-brain barrier and treat brain metastasis from V600E mutant NSCLC [9].

To the best of our knowledge, we believe this is the first case report of a rare molecular subtype of LAC with BRAF V600E mutation, initially presenting with visual loss due to a skull base metastasis collision tumour with a pituitary adenoma.

## Conclusions

Collision tumours involving pituitary adenoma and sella turcica metastasis from lung adenocarcinomas are rare. Our findings show a rapid response to endoscopic resection in a patient who presented unusually with loss of vision secondary to a metastatic pulmonary adenocarcinoma and pituitary adenoma. The BRAF V600E mutation identified in the tumour highlights the importance of routine molecular profiling in lung adenocarcinomas. Dual BRAF-MEK inhibitors should be explored in patients with LAC with BRAF V600E mutation.
